# Influence of rootstocks on growth, yield, fruit quality and leaf mineral element contents of pear cv. ‘Santa Maria’ in semi-arid conditions

**DOI:** 10.1186/0717-6287-47-71

**Published:** 2014-12-16

**Authors:** Ali Ikinci, Ibrahim Bolat, Sezai Ercisli, Ossama Kodad

**Affiliations:** Horticulture Department, Agriculture Faculty, Harran University, 63330 Sanliurfa, Turkey; Horticulture Department, Agriculture Faculty, Ataturk University, 25240 Erzurum, Turkey; Department of Pomology, National School of Agriculture, Meknes, Morocco

**Keywords:** TCSA, BA-29, MA, MC, Pear seedlings, *Pyrus communis* L, *Cydonia oblonga* L

## Abstract

**Background:**

Rootstocks play an essential role to determining orchard performance of fruit trees. *Pyrus communis* and *Cydonia oblonga* are widely used rootstocks for European pear cultivars. The lack of rootstocks adapted to different soil conditions and different grafted cultivars is widely acknowledged in pear culture. *Cydonia* rootstocks (clonal) and *Pyrus* rootstocks (seedling or clonal) have their advantages and disadvantages. In each case, site-specific environmental characteristics, specific cultivar response and production objectives must be considered before choosing the best rootstock. In this study, the influence of three Quince (BA 29, Quince A = MA, Quince C = MC) and a local European pear seedling rootstocks on the scion yield, some fruit quality characteristics and leaf macro (N, P, K, Ca and Mg) and micro element (Fe, Zn, Cu, Mn and B) content of ‘Santa Maria’ pear (*Pyrus communis* L.) were investigated.

**Results:**

Trees on seedling rootstock had the highest annual yield, highest cumulative yield (kg tree^−1^), largest trunk cross-sectional area (TCSA), lowest yield efficiency and lowest cumulative yield (ton ha^−1^) in the 10^th^ year after planting. The rootstocks had no significant effect on average fruit weight and fruit volume. Significantly higher fruit firmness was obtained on BA 29 and Quince A. The effect of rootstocks on the mineral element accumulation (N, K, Ca, Mg, Fe, Zn, Cu, Mn and B) was significant. Leaf analysis showed that rootstocks used had different mineral uptake efficiencies throughout the early season.

**Conclusion:**

The results showed that the rootstocks strongly affected fruit yield, fruit quality and leaf mineral element uptake of ‘Santa Maria’ pear cultivar. Pear seedling and BA 29 rootstock found to be more prominent in terms of several characteristics for ‘Santa Maria’ pear cultivar that is grown in highly calcareous soil in semi-arid climate conditions. We determined the highest N, P (although insignificant), K, Ca, Mg, Fe and Cu mineral element concentrations on the pear seedling and BA 29 rootstocks. According to the results, we recommend the seedling rootstock for normal density plantings (400 trees ha^−1^) and BA 29 rootstock for high-density plantings (800 trees ha^−1^) for ‘Santa Maria’ pear cultivar in semi-arid conditions.

## Background

Pear (*Pyrus communis* L.) is one of the major fruit in the world and grown well in temperate zones of both hemispheres. The world pear production is about 24 million tons and China is main producer shared with 68% of the world’s pear production and followed by the USA (3.3%), Argentina (3.0%), Italy (2.7%) and Turkey (1.9%) [1].

In the commercial pear production, various vegetatively propagated quince and pear rootstocks and generative pear rootstocks have been used. In Turkey, the most common rootstock used for pear cultivars is wild pear seedlings with approximately 85-90% due to their tolerance to lime induced iron chlorosis, easy propagation and well graft-compatible with pear cultivars. They also grow vigorously in loamy wet soil and unfavourable conditions [2, 3]. The selection of clonal quince (*C. oblonga*), such as Quince A (MA), Quince C (MC) and BA 29 in Europe, or of clonal *Pyrus communis* L., such as ‘Old Home’ × ‘Farmingdale’ (OHF) in the USA or in South Africa, as substitutes for pear seedling rootstock, have clearly improved the precocity, productivity and quality of some European pear cultivars [2, 3].

The rapid developments fruit tree nursery technology and rootstock research and introduction of new clonally propagated rootstocks opened in new area in fruit science [4, 5]. For this reason more recently modern pear orchards with different modern training systems to start establish with use of clonal quince (*Cydonia oblonga* L.) rootstocks such as Quince A, Quince C and BA 29 in Turkey. These clonal rootstocks with dwarfing characteristics well reported to increase precocity and fruit quality, especially in the high intensity modern orchards and thus gained more importance [6–8].

Previously, several reports have been documented the relationships between various physiological parameters of pear cultivar/various rootstocks combinations [6–10]. These relationships are important from a horticultural point of view, because they provide a basis for selecting the best graft combination for particular environmental conditions and high fruit quality. Selection of an appropriate graft combination is crucial for the production of deciduous orchard species, because the scion–rootstock interaction influences water relations, leaf gas exchange, plant size, blossoming, timing of fruit set, fruit quality and yield efficiency [10–14]. Different rootstocks have also showed different mineral uptake efficiencies [15]. Leaf mineral element analysis is an effective method for fruit tree nutrient diagnosis and fertilization calculation. Similarly, symptoms of iron deficiency could be mitigated by analyses of mineral leaf composition prior to harvest [16, 17]. Moreover, accurate water and fertilizer management are essential in the highly intensive orchard systems to enable the manipulation of both reproductive and vegetative development, to ensure the possibility obtaining higher fruit quality with longer storage potential and to reduce pollution and costs [7].

Southern Anatolia region in Turkey is characterized by fertile soil and semi-arid conditions favorable for growing of subtropical and temperate fruits. More recently in particular the use of clonally propagated dwarf rootstocks for temperate fruit species including pear are widespread in this region. However, the knowladge of specific rootstock effects on specific scion cultivars is of utmost importance to get maximum benefits from the enterprises.

Thus, this study is mainly focused on the effects of various clonal and seedling rootstocks on the main production traits of scion pear cv. ‘Santa Maria’. Although this cultivar has already grown commercially in Southern Anatolia region, there is a need to increase the production of this fruit.

## Results and discussion

**Figure 1:**
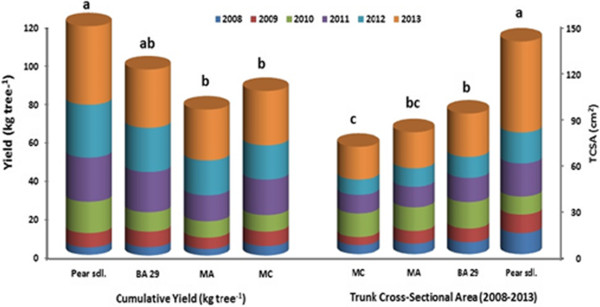
**Cumulative yield (kg tree** ^**−1**^ **) and trunk cross-sectional area (TCSA) of ‘Santa Maria’ pear cultivars grown on different rootstocks.** Different letters denote significant differences between means, according to Duncan’s multiple range test, *P*< 0.05.

As indicated in Figure 1, there were statistically significant differences among rootstocks in terms of cumulative yield and Trunk Cross Sectional Area (TCSA) (p < 0.05). TCSA of ‘Santa Maria’ pear trees were significantly affected by rootstocks (p > 0.05, Figure 1) and TCSA were found to be highest when ‘Santa Maria’ grafted on seedling rootstock and followed by BA 29, and the lowest one obtained from MA and MC (Figure 1). ‘Santa Maria’ pear trees grafted on the *Pyrus communis* seedling rootstock gave the highest annual yield between the years of 2008-2013, compared to the other three clonal Quince rootstocks (BA 29, MA, MC) (Figure 1). Similar to the annual yield, cumulative yield was significantly higher for ‘Santa Maria’ grown on seedling rootstock than the other rootstocks tested during 2008 through 2013 (Figure 1).

**Figure 2:**
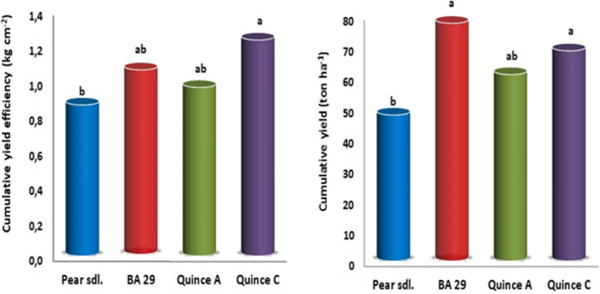
**Cumulative yield efficiency (kg cm** ^**−2**^ **) and cumulative yield (ton ha** ^**−1**^ **) of ‘Santa Maria’ pear cultivars grown on different rootstocks.** Different letters denote significant differences between means, according to Duncan’s multiple range test, *P*< 0.05.

Cumulative yield efficiency (CYE) significantly affected by rootstocks (p < 0.05), with the highest was observed on MC and the lowest ones on seedling rootstock (Figure 2). There was an opposite trend between CYE and TCSA. After 10 years, the cumulative yield on BA 29 was 77.36 t ha^−1^ and MC was 68.41 t ha^−1^ considerably higher than on MA (60.69 t ha^−1^) and 47.52 t ha^−1^ on seedling rootstock (Figure 2). Castro and Rodriguez [11] found that yield of the ‘Abbe Fetel’ and ‘Conference’ pear cultivars grafted on pear seedling was higher than the quince selections MA and BA 29. It was reported that pear cultivar ‘Conference’ grafted on BA 29 rootstocks had higher trunk circumference in comparison to MA and MC quince rootstocks [12]. Haak et al. [13] reported that TCSA value of ‘Suvenirs’ pear cultivar that is grafted onto different *Pyrus* and *Cydonia* rootstocks was the highest on *Pyrus* rootstock and the lowest on on MC rootstock, 5 years after plantation. Sotiropoulos [14] reported that production efficiency of ‘William’s BC’ was highest when grafted on PI 27 (local quince seedlings), intermediate on MA and and lowest on *P. communis.*

Rootstock had a significant effect on tree size, as reflected by TCSA measurements. From planting of trees up to 10 years, although, trees on vigorous seedling rootstocks can have higher yield than those on dwarfing ones due to their greater size, this superiority may not hold for yield efficiency which is production per unit of growth. Yield efficiency does not seem to be clearly related to rootstock vigour [18]. Wertheim [19] reported that MC and BA 29 rootstocks showed higher yield efficiency than OH (Old Home) 11, OH 20, OH 33 and OHF (Old Home x Farmingdale) 333 rootstocks. As regards to yield efficiency the encountered data agree with Loreti et al. [12] and Giacobbo et al. [20] who analyzed different pear rootstocks and verified that the high yield efficiency is not always directly related to high production, once the rootstocks that increased production did not improve yield efficiency. These results are in general agreement with the findings of the researchers mentioned above.

There seemed to be no significant differences between rootstocks in terms of average fruit size and average fruit volume of ‘Santa Maria’ pear (Table 1). However, trees on MC had higher fruit size and average fruit volume than those on MA, BA 29 and seedling rootstock. Fruit flesh firmness, Soluble Solid Content (SSC) and Titratable Acidity (TA) were significantly affected by rootstocks (p < 0.05). Trees on BA 29 and MA had higher fruit flesh firmness than those on MC and seedling rootstocks. Fruit SSC was the highest on seedling rootstock, whereas fruit TA concentration was the highest on BA 29 rootstock (Table 1).

**Table 1:** **Some quality characteristics of “Santa Maria” fruit, as influenced by rootstocks (2012-2013)**

Rootstock	Average fruit weight	Average fruit volume	Fruit flesh firmness (lb)	SSC	TA
	(g)	(cm ^3^)		(%)	(%)
Pear seedling	265.49	291.20	18.98b	15.60a	0.20c
BA 29	277.55	301.63	21.32a	14.60b	0.26a
MA	279.20	316.80	20.76a	14.90ab	0.24ab
MC	290.37	325.80	19.47ab	15.25ab	0.22bc
*Significance*	ns	Ns	*	**	**

ns, *and **Nonsignificant or significant at *P* ≤0.05 or 0.01, respectively.

In our study, mean fruit weight value was between 265 and 290 g and mean fruit volume was 291-325 cm^3^ in ‘Santa Maria’ trees on all rootstocks (Table 1). Previous studies on pear reported mean fruit weight value as 147 and 190 g in ‘Santa Maria’ pear cultivar [21, 22]. Fruit weight values that we obtained from ‘Santa Maria’ cultivar are approximately 100 g higher than the fruit weight values obtained in previous studies. In this study the highest mean fruit weight and fruit volume values were obtained from MC rootstock, while the lowest values were obtained from the trees on seedling. Rootstock can effect directly or indirectly pear fruit size and weight. This effect has been shown by some *Pyrus* rootstocks (OHF 33, OHF 333), which resulted in smaller fruits than the quince rootstocks (BA 29, MC) with which they were compared in spite of lower fruit densities [14]. Wertheim [19] showed that quince rootstocks can produce larger fruit than *Pyrus* rootstocks. In this study, rootstocks did not have a statistically significant effect on fruit size. In our study, we obtained higher cumulative yield values from the trees on seedling rootstock, which is a strong rootstock, while correspondingly lower average fruit weight values were obtained from these rootstocks. The fact that the trees on seedling, which gave a higher yield and more fruit number can be considered as one of the reasons for lower mean fruit weight when compared to other rootstocks.

In this research, the highest fruit firmness (21.32 and 20.76 lb, respectively) was recorded on BA 29 and MA rootstocks whereas, the lowest (18.98 lb) on seedling rootstock (Table 1). Erdem and Öztürk [21] found fruit firmness as 18.6-21.36 lb in ‘Santa Maria’ pear cultivar. Fruit firmness values we obtained in our study are similar to the values reported by this researchers. Fruit firmness is one of the most important maturity and quality parameters. Fruit firmness decreases as the maturity level of fruits increase. Nutrient elements taken from the soil or given to the plant from the leaves can reduce fruit firmness.

It was found that the trees on *Pyrus* seedling rootstock had the highest SSC (15.60%) and the lowest (0.20%) TA content (Table 2). In contrast, Sotiropoulos [14] reported that SSC of fruits of ‘William’s BC’ pear cultivar grafted on BA 29 and MA were significantly higher in comparison to *Pyrus* seedling.

**Table 2:** **Seasonal variation in N, P, K, Ca, and Mg foliar concentration of Santa Maria’ pear cultivar grown on different rootstocks**

Rootstock	N (%)			P (%)			K (%)			Ca (%)			Mg (%)		
	30 DAFB	60 DAFB	90 DAFB	30 DAFB	60 DAFB	90 DAFB	30 DAFB	60 DAFB	90 DAFB	30 DAFB	60 DAFB	90 DAFB	30 DAFB	60 DAFB	90 DAFB
Pear sdl.	2.38 a	2.14 a	2.05 a	0.18	0.17	0.17	1.94 a	1.88 a	1.53 a	1.66 a	1.84 a	1.88 a	0.40 a	0.45 a	0.47 a
BA 29	2.25 ab	2.11 ab	1.98 ab	0.22	0.15	0.18	1.89 a	1.85 a	1.42 ab	1.51 ab	1.64 a	1.67 a	0.38 a	0.44 a	0.45 a
Quince A	2.19 b	1.99 c	1.87 b	0.18	0.13	0.15	1.53 b	1.39 b	1.27 b	1.34 b	1.37 b	1.46 b	0.37 ab	0.40 ab	0.42 ab
Quince C	2.17 b	2.03 bc	1.97 ab	0.19	0.11	0.14	1.58 b	1.45 b	1.44 ab	1.11 c	1.30 b	1.38 b	0.22 b	0.34 b	0.36 b
*Significance*	**	**	**	Ns	Ns	Ns	***	***	**	***	***	***	**	*	*

Values are the mean for 2012 and 2013.

Norms: Heckman [36] N (2.20-2.80%), P (0.11-0.25%), K (1.00-2.00%), Ca (1.00-1.50%) and Mg (0.25-0.50%).

ns, *, **and ***Nonsignificant or significant at *P* ≤ 0.05, 0.01 or 0.001, respectively.

SSC content increases with increasing fruit ripening, whereas the longer ripening period TA content shows the decrease [23]. Ozturk et al. [21] reported SSC and TA contents for ‘Santa Maria’ cultivar as 12.50% and 0.48% respectively. These differences in chemical composition of the same pear cultivar may be due to different soil type and fertility, different genetic crop loads, tree age, differences in rootstocks, differences in ecology, fertilization, irrigation level and differences in the harvest period.

SSC content of the fruits on *Cydonia oblonga* species rootstocks was determined to be lower than the fruits on *Pyrus* seedling rootstocks (Table 1). Some authors reported that Fe chlorosis can affect some growth parameters such as skin color, fruit firmness, titratable acidity, soluble solid content, organic acids, carbohydrates composition, vitamins and phenolics compounds [24, 25]. This may be linked to the photosynthetic activity of plants, because CO_2_ assimilation of chlorotic leaves and carbohydrates allocation in fruits were negatively affected [26].

The effect of rootstocks on leaf mineral element concentrations of ‘Santa Maria’ was statistically significant for N, K, Ca, Mg, Fe, Zn, Mn, Cu and B (Tables 2 and 3). The concentration of Ca, Mg, Mn and Cu in leaves increased from 30 to 90 days after full bloom (DAFB). Leaf P concentration decreased from 30 to 60 DAFB, and then increased thereafter, whereas leaf N, K, Fe, Zn, and B concentrations decreased from 30 to 90 DAFB. ‘Santa Maria’ on seedling rootstock had the highest leaf N (2.05%), K (1.53%), Fe (50.56 μg g^−1^), Cu (21.80 μg g^−1^), and B (16.43 ppm) concentrations at the 90 DAFB than other rootstocks. The highest leaf Ca (1.88% and 1.67%) and Mg (0.47% and 0.45%) concentrations were shown on seedling and BA 29 rootstocks, respectively. Among the rootstocks, ‘Santa Maria’ pear cultivar had the highest leaf Zn (26.7 and 24.6 μg g^−1^, respectively) concentrations on MC and MA, whereas the highest leaf Mn (75.10 ppm) concentration only on MC.

**Table 3:** **Seasonal variation in Fe, Zn, Mn, Cu, and B foliar concentration of Santa Maria’ pear cultivar grown on different rootstocks**

Rootstock	Leaf Fe (μg g ^−1^)			Leaf Zn (μg g ^−1^)			Leaf Mn (μg g ^−1^)			Leaf Cu (μg g ^−1^)			Leaf B (μg g ^−1^)		
	30 DAFB	60 DAFB	90 DAFB	30 DAFB	60 DAFB	90 DAFB	30 DAFB	60 DAFB	90 DAFB	30 DAFB	60 DAFB	90 DAFB	30 DAFB	60 DAFB	90 DAFB
Pear sdl.	72.3 a	61.6 a	50.6 a	27.9 c	20.8 b	16.2 c	37.8 d	38.5 d	44.9 d	14.8 a	18.0 a	21.8 a	25.8 b	19.8 a	16.4 a
BA 29	69.2 b	60.1 a	46.7 b	32.7 b	24.9 b	21.3 b	43.9 b	46.0 c	52.2 c	15.8 a	15.9 b	17.8 b	27.4 a	7.3 c	6.7 c
Quince A	57.9 d	50.4 c	40.7 c	37.2 a	30.9 a	24.6 a	42.9 c	48.8 b	59.2 b	10.3 c	16.0 b	17.0 c	17.4 d	9.2 b	8.2 b
Quince C	64.9 c	55.7 b	41.2 c	40.8 a	32.7 a	26.7 a	45.8 a	68.8 a	75.1 a	12.9 b	14.4 c	15.7 d	21.0 c	9.5 b	7.9 b
*Significance*	***	***	***	**	**	**	***	***	**	***	***	***	***	***	***

Values are the mean for 2012 and 2013.

Norms: Heckman [36] Fe (60-250 μg g^−1^), Zn (25-200 μg g^−1^), Mn (30-100 μg g^−1^), Cu (5-20 μg g^−1^) and B (20-70 μg g^−1^).

**and ***Significant at *P* ≤ 0.01 or 0.001.

In our study, we found significant differences in the mineral concentrations of leaves of ‘Santa Maria’ pear trees grafted on BA 29, MA, MC, and seedling rootstocks (Tables 2 and 3). Other researchers have also reported significant rootstock effects on scion leaf mineral nutrients concentrations of some fruit trees under different environmental conditions [27–29].

Based on our findings of macro elements, Ca and Mg levels generally showed an increase from 30 DAFB to 90 DAFB, while N and K concentrations generally showed a decrease (Table 2). P concentration was found to show a decrease between 30 DAFB - 60 DAFB and then to show an increase between 60 DAFB - 90 DAFB (Table 2). Belkhodja et al. [30] found that N, P and K concentrations were decreased in leaves of peach trees from 60 to 120 DAFB, whereas Ca and Mg concentrations were increased from 60 to 120 DAFB. The results we have obtained from this study's findings are in agreement with Belkhodja et al. [30]. In our study, the results that we have obtained similarly, in another study, the concentration of most nutrients in leaves decreased as the growing season progressed, with only that of Ca, Mg, and Mn showing an increase [31].

Leaf Ca and Mg concentration was higher (at the 90 DAFB) on seedling and BA 29, and lower on MA and MC (Table 2). Trees grafted on MA and MC appears to have the lowest leaf macronutrient concentration. The same effect on the leaf concentration was also found in lower vigour rootstocks in apple [29, 32, 33]. Several researchers have shown that scion leaves of trees on more vigorous rootstocks have higher mineral (K, Mg) content than those on size-controlling rootstocks [32, 34]. Previously dwarf rootstock is rated as sensitive to Ca and K deficiencies, which is in agreement with our result [34].

It can be concluded that dwarfing rootstocks were less effective than others in terms of uptake of some macronutrients from root medium. Their nutrient uptake capacity is less due to poor root volume in the soil [35]. Differences in nutrient concentrations among rootstocks can also be explained with the structure of root systems, deviations of root cation exchange capacities, rhizosphere pH, characteristics of root exudates etc. [35, 36].

Previously in pear leaf sample collection time as 35 to 70 DAFB period is reported more suitable [37], some reported that the collection time is 120 DAFB more suitable [36]. Leaf analysis results of ‘Santa Maria’ pear trees in 90 DAFB period showed that N, Fe, Zn and B concentrations in some rootstocks (Tables 2 and 3) were much lower than reference values [36]. N and Fe were found to be particularly lower in the leaves of the trees on MA and MC rootstocks.

Leaf Fe concentration was found to be lower than the threshold value (60 μg g^−1^) reported by Heckman [36] starting from 30 DAFB in pear trees on *Cydonia* rootstock and starting from 60 DAFB in trees on seedling rootstock (Table 3). Leaf Fe concentration of ‘Santa Maria’ trees on all rootstocks has fallen well below the reference value at 90 DAFB. Pear is the leading fruit species with the most commonly Fe chlorosis seen in all fruit species [38]. Significantly high soil pH and lime ratio in soil in the orchard where the study was carried out is a major cause of the decrease of Fe concentration in ‘Santa Maria’ leaves starting from 30 DAFB.

Iron chlorosis increased markedly leaf K concentrations, and only slightly leaf N, Mg and Mn concentrations, whereas leaf P, Cu and Zn were not affected much by chlorosis [30]. Our pear leaf results agree with the leaf nutrient concentrations time courses obtained by Belkhodja et al. [30] in peach grown in Zaragosa (Spain).

Zn concentration in ‘Santa Maria’ leaves began to decrease starting from 30 DAFB on all rootstocks (Table 3). Zn concentration in the leaves of ‘Santa Maria’ on seedling and BA 29 rootstocks fell below the reference value (25-200 μg g^−1^) at 60 DAFB. This decrease continued at 90 DAFB. It was found that Zn concentration of ‘Santa Maria’ leaves (26.7 μg g^−1^) was above the reference value only on 90 DAFB date on MC rootstock. No visual sign of Zn deficiency was observed on ‘Santa Maria’ leaves until 90 DAFB stage in 2012 and 2013. Erdem and Ozturk [22] reported that ‘Santa Maria’ cultivar is more resistant to Zn deficiency than ‘Akça’ and ‘Deveci’ cultivars and that; it used the existing zinc in soil better. Swietlik [39] reported that Zn deficiency is common in fruit trees that grow in alkaline soils with high pH content. It can be stated that high pH level of the soil is a reason for Zn concentration level below the threshold value in ‘Santa Maria’ leaves.

Trees on seedling rootstock had higher leaf B concentrations than those on other rootstocks (Table 3). Lombard and Westwood [40] reported that pear seedling rootstocks have a higher B uptake than quince rootstocks.

## Conclusion

The effects of rootstocks on fruit yield and quality and mineral element uptake of ‘Santa Maria’ pear cultivar showed variations. Pear seedling and BA 29 rootstock became prominent in terms of several characteristics for ‘Santa Maria’ pear cultivar that is grown in highly calcareous soil in semi-arid climate conditions. The trees on seedling rootstock were found to have higher values than other rootstocks in terms of annual yield, cumulative yield and TCSA value. 77.4 ton ha^−1^ yield was obtained from 10 year old ‘Santa Maria’ trees grafted on BA 29 rootstock at a density of 800 trees ha^−1^ in 2008-2013 periods. In the orchard used in the study, soil pH was significantly high. The highest N, P (although insignificant), K, Ca, Mg, Fe and Cu concentrations were determined in the trees on pear seedling and BA 29 rootstocks. The lowest leaf Fe concentrations in pear trees were determined in the trees on MA and MC rootstocks. Leaf Fe concentrations of the trees on these rootstocks began to decrease from 30 DAFB and began to fall below the critical threshold after this date. According to the results obtained from this study, we recommend the seedling rootstock for normal density plantings (400 trees ha^−1^) and BA 29 rootstock for high density plantings (800 trees ha^−1^) at the ‘Santa Maria’ orchards in semi-arid conditions.

## Methods

### Site description

**Figure 3:**
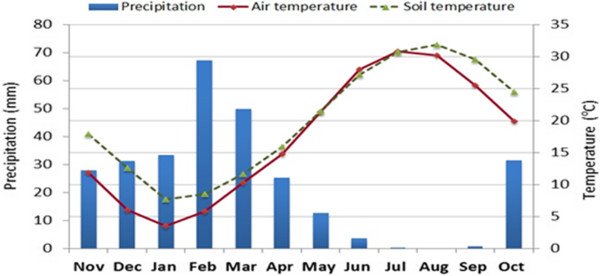
**Average monthly precipitation, air and soil temperature between 2008 and 2013.**

The experiment was carried out at the Harran University Pome Fruit Research Station in Sanliurfa, Turkey (37°10' N, 38°59' E; alt. 520 m) during 2008-2013. Sanliurfa province has semi-arid climate features with cold and wet during the winter and very hot and dry in the summer seasons. During the experiment, the air temperatures were in average 29.9°C in summer and 9.4°C in winter, while annual precipitation ranged between 355-447 mm, mainly concentrated between the months of November and April (Figure 3). The average relative humidity is at the level of 52.2%. Relative humidity is the highest (66%) ratio in January and in July is the lowest (36%) level. The orchard was established in a calcareous (21.5% total carbonates and 10.7% active lime), alkaline and clay-loam textured soil*.* The physical and chemical characteristics of the soil were clay 58.5%, silt 18.5% and sand 21%, with the low level of organic matter (1.16%), pH 7.92 (in 1M KCl), and optimum concentrations of available P (80 mg kg^−1^), K (160 mg kg^−1^), Mg (50 mg kg^−1^), and Fe (DTPA-extractable Fe:1.45 mg kg^−1^) in the top soil layer (0–40 cm).

### Plant material and experimental design

‘Santa Maria’ pear trees were planted in December 2004 with 1-year-old scions. The following rootstocks were tested: Local pear seedling (*Pyrus communis* L.), clonal MA, MC and BA 29 (*Cydonia oblonga* Mill.). The experiment was laid out in a randomized complete-block design with three blocks, each consisting of three rows of trees. There were 15 trees in each row. Each experimental plot contained seven trees in each row. Data were collected from the five central trees in each row, using the remaining trees as guards. Trees on pear seedling rootstocks (hereafter referred to as “seedling rootstocks”) were planted at 5 × 5 m (400 trees ha^−1^) and trees on the *Cydonia oblonga* variety rootstocks were planted at 5 × 2.5 m (800 trees ha^−1^) distance and trained as a central leader system.

### Cultural treatments

Irrigation of the orchard was carried out using a computerized drip irrigation system. Irrigation frequency was two times per week from May to October each season according to regional recommendations using class-A pan. Each treatment (tree) received the same total amount of water in each season. All treated trees were similarly fertigated with essential minerals using the fertigation method. No foliar application of nutrients was made to these trees. Thinning of flowers or fruitlets was not carried out during the experiment. Weed, disease, and insect control was managed using the practices that were commonly used for commercial production, and all the treatments were under the identical management. A copper spray was put on at budbreak to protect the trees from fireblight.

### Data collection on growth, yield, fruit characteristics

Trunk diameter 20 cm above the graft union was measured with digital callipers in December each year. The average of two readings (north-south and east- west) was converted to trunk cross-sectional area (TCSA) for analysis. Annual yields, yield efficiency (yield/TCSA), cumulative yield and cumulative yield efficiency (cumulative yield/TCSA in 2013) were calculated. Cumulative fruit yield efficiency (CYE) was expressed as kg cm^−2^ [41].

Fruit yield was determined each year by harvesting five central trees from each plot in September. Fruit firmness, soluble solids concentration (SSC), and titratable acidity (TA) of fruits at harvest were determined using a randomly selected sample of 20 fruits for each plot. Fruit yield per tree and average fruit weight were measured at fruit harvest in September. Fruit firmness was measured individually on two opposite faces of peeled fruits by using Effegi penetrometer (model. FT–327; McCormick Fruit Tech, Yakima, WA) with an 8 mm diameter tip and expressed in terms of lb force. The SSC was determined with an Atago Palette Series Model PR-101a digital refractometer (Atago Co. Ltd., Tokyo, Japan) at 22°C in the juice squeezed from the fruit homogenate (expressed as ^o^Brix). TA was determined by titrating the fruit homogenate with 0.1 N NaOH to pH 8.1. The TA results represented malic acid content expressed as a percentage. All analyses were performed according to standard methods [42].

### Data collection on leaf mineral elements content

Leaf mineral concentrations were determined in 2012 and 2013. Leaf sampling was done at 30, 60 and 90 days after full bloom (DAFB). Each leaf sample consisted of 50 new but fully developed midterminal leaves from current-year shoots at 150 cm above the ground in the tree canopy [28, 36]. Collected leaves were immediately packed into polyethylene bags and transported to the laboratory in a portable refrigerator. The leaf samples were washed in tap water, 0.1 mol L^−1^ of HCl and deionized water then dried in a forced air drying oven at 65°C for 48 h to constant weight. Leaves were ground to pass a 40 mesh screen and stored in an oven at 60°C until analysis. One g of dried ground leaf sample dry ashed at 550°C for 5 h. The ash was then dissolved in 0.1 N HCl. Analyses were performed by a colorimetric method for P (phospho-vanadate reaction), Atomic Emission Spectrometry for K and Na, and Atomic Absorption Spectrometry (Perkin-Elmer 1100 B, Norwalk, CT) for Ca, Mg, Fe, Mn, Zn and Cu. Nitrogen was determined by the Kjeldahl procedure. Leaf boron (B) concentration was determined by spectrophotometry using the Azomethine-H method, in extracts obtained from leaf ashes (oven digestion) according to procedure described by Kacar [43]. Each determination was replicated three times. The results were expressed on a dry matter basis: % for macro (N, P, K, Ca and Mg) and mg kg^−1^ for microelements (Fe, Mn, Cu, Zn and B).

### Statistical analysis

Analyses of variance were performed on all the data collected. Percentage data were subjected to arcsine transformation before analysis, to provide a normal distribution. Differences between the means were ascertained with Duncan’s multiple range tests, using the SAS software package (SAS Institute, Cary, NC). The mean values for the combinations labeled with the same letters do not significantly differ at the significance level α = 0.05.
